# Dietary diversity moderates household economic inequalities in the double burden of malnutrition in Tanzania

**DOI:** 10.1017/S136898002400106X

**Published:** 2024-05-16

**Authors:** Sanmei Chen, Yoko Shimpuku, Takanori Honda, Dorkasi L Mwakawanga, Beatrice Mwilike

**Affiliations:** 1 Global Health Nursing, Graduate School of Biomedical and Health Sciences, Hiroshima University, Hiroshima 734-8553, Japan; 2 Center for Cohort Studies, Graduate School of Medical Sciences, Kyushu University, Fukuoka, Japan; 3 Department of Community Health Nursing, School of Nursing, Muhimbili University of Health and Allied Sciences, Dar es Salaam, Tanzania

**Keywords:** Malnutrition, Dietary diversity, Tanzania, Household wealth

## Abstract

**Objective:**

Improved food availability and a growing economy in Tanzania may insufficiently decrease pre-existing nutritional deficiencies and simultaneously increase overweight within the same individual, household or population, causing a double burden of malnutrition (DBM). We investigated economic inequalities in DBM at the household level, expressed as a stunted child with a mother with overweight/obesity, and the moderating role of dietary diversity in these inequalities.

**Design:**

We used cross-sectional data from the 2015–2016 Tanzania Demographic and Health Survey.

**Setting:**

A nationally representative survey.

**Participants:**

Totally, 2867 children (aged 6–23 months) and their mothers (aged 15–49 years). The mother–child pairs were categorised into two groups based on dietary diversity score: achieving and not achieving minimum dietary diversity.

**Results:**

The prevalence of DBM was 5·6 % (s d = 0·6) and significantly varied by region (ranging from 0·6 % to 12·2 %). Significant interaction was observed between dietary diversity and household wealth index (*P*_for interaction_ < 0·001). The prevalence of DBM monotonically increased with greater household wealth among mother–child pairs who did not achieve minimum dietary diversity (*P*_for trend_ < 0·001; however, this association was attenuated in those who achieved minimum dietary diversity (*P*_for trend_ = 0·16), particularly for the richest households (*P* = 0·44). Analysing household wealth index score as a continuous variable yielded similar results (OR (95 % CI): 2·10 (1·36, 3·25) for non-achievers of minimum dietary diversity, 1·38 (0·76, 2·54) for achievers).

**Conclusions:**

Greater household wealth was associated with higher odds of DBM in Tanzania; however, the negative impact of household economic status on DBM was mitigated by minimum dietary diversity.

Countries worldwide are now experiencing a fast-evolving and more complex nutrition paradigm^(1)^. Instead of focusing on a single side of malnutrition, combating all forms of malnutrition is among the top priorities of the United Nations Decade of Action on Nutrition and the Sustainable Development Goals (SDG, Target 2·2)^(2,3)^. Undernutrition and overweight or obesity have been historically addressed as separate challenges affecting distinct populations with contrast risk factors^(4)^. However, the changing global nutrition reality is that these two distinct forms of malnutrition frequently coexist within individuals, households and populations, with common mechanisms (e.g. economic inequalities^(5)^) and consequences on health^(4)^. This growing recognition in the global health community forms the basis of the emerging concept of double burden of malnutrition (DBM)^(6,7)^. This global double burden of undernutrition and obesity and its great developmental and socio-economic impact have been recognised as serious and lasting in low- and middle-income countries (LMIC) undergoing rapid nutrition transition^(8–10)^; however, they have not yet been examined extensively.

Tanzania is experiencing improved food availability as its economy is growing rapidly. Economic transition with an increased average household income enables more households to purchase more food^(11)^, which potentially improves undernutrition. However, the rates of decline in undernutrition in children under age five in Tanzania (e.g. stunting from 34·4 % in 2014 to 31·8 % in 2018) are still too slow to meet the SDG targets by 2030^(12)^. Even worse, the prevalence of child underweight increased from 13·7 % in 2014 to 14·6 % in 2018^(12)^. Simultaneously overweight and obesity is rapidly growing, affecting over 30 % of Tanzanian women aged 15–49 years^(12)^, perhaps mainly due to major reductions in physical activities at work, transportation and home and increased consumption of cheap ultra-processed fast food and beverages^(13,14)^. The coexistence of persisting undernutrition and rising obesity may increase DBM in Tanzania^(15)^.

DBM at the household level is defined as multiple family members affected by different forms of malnutrition^(8)^. Household-level DBM varies between countries and often arises in lower-middle-income countries including Tanzania^(14)^. Evidence showed that the prevalence of the total household-level DBM ranged between 3 % and 35 % across 126 LMIC, with child stunting and maternal overweight/obesity being the most prevalent DBM type (ranging between 1 % and 24 %)^(14,16)^. Household-level DBM has been shown to be primarily driven by socio-economic inequalities; however, the effect of household economic status on DBM is heterogeneous^(17–21)^. In poorer LMIC higher household economic levels were linked to increased odds of DBM, while in richer LMIC lower household economic levels were associated with higher odds of DBM^(22)^. In Tanzania, it remains uncertain how household economic inequalities are associated with DBM. A univariate analysis in Tanzania reported a 1·4 times higher crude likelihood of DBM among richer households; however, this study did not account for important household characteristics such as place of residence when quantifying this association^(15)^.

Dietary diversity, a practical and valid indicator of nutrient/micronutrient adequacy in assessing maternal and child nutrition in LMIC, is hypothesised to be an underrated action target for addressing DBM^(23,24)^. However, the role of dietary diversity in this association remains uncertain^(17–22)^. Poor dietary diversity remains prevalent in Africa^(25,26)^, especially among populations with diets based on starchy staples like Tanzanians^(27,28)^. Generally, dietary diversity increases as household income increases^(28)^, thus it may mediate the beneficial effects of household income on improving nutrient adequacy and diet-related health outcomes^(29)^. Paradoxically, in emerging economies and African countries, economic growth or family income has not yet efficiently improved dietary diversity^(26,30,31)^, but worsened nutrition-related health outcomes^(5,22,31)^. This is partly because other factors, such as cultural preferences^(27)^, lack of nutrition knowledge^(27)^, and unimproved food systems^(32)^, contribute significantly^(30)^. We assumed that the unimproved dietary diversity may play a moderating role in attenuating the potential adverse impact of household wealth on DBM in Tanzania.

In this study, we aimed to investigate household economic inequalities in DBM, expressed as child stunting and maternal overweight/obesity, and the moderating role of dietary diversity in these inequalities. We hypothesised that the association between household wealth and DBM may be weaker among mother–child pairs with a higher dietary diversity.

## Methods

### Data

We obtained cross-sectional data from the 2015–2016 Tanzania Demographic and Health Survey, provided by the United States Agency for International Development^(33)^. The data were from nationally representative household surveys of girls and women of productive age (15–49 years) and their children born in the five years preceding the survey, using a stratified two-stage cluster sampling method. This sampling technique allowed each household to have an equal probability of participating in the survey. In the present study, we used the dataset for children under the age of five and their mothers. This dataset provides anthropometric information for each child, as well as the characteristics of the mother and household (*n* 10 233)^(34)^. For the analysis, we included children aged 6–23 months old (*n* 3320), who were recommended by the WHO/UNICEF as key targets for assessing infant and young child feeding practices using diet quality indicators such as dietary diversity^(35)^. We excluded children who were not alive (*n* 137), children who were not living with their mothers (*n* 47) and children with height missing values (*n* 55). Moreover, we excluded mothers who were pregnant (*n* 207) and those with missing values of weight or height (*n* 7). Our final sample consisted of 2867 mother–child pairs (weighted sample size: *n* 2850) (see online supplementary material, Supplemental Fig. S1).

### Double burden of malnutrition

Anthropometric data (weight and height) were collected based on the standard procedures from the WHO^(34,36,37)^. Weight was measured with an electronic SECA 874 flat scale in 0·1 kg increments^(34)^. For very young children, the mother or caretaker was weighed first and then weighed again while holding the child^(34)^. The weight scale allowed the mother’s stored weight to be deducted and showed the child’s weight on the display. Height was measured with a short measuring board in a standing position, while children younger than 24 months or shorter than 85 cm were measured lying down on the board (recumbent length)^(34)^.

DBM can occur in different scenarios, including when a child is both stunted and overweight, when a child is wasted with a mother who is overweight/obese, when a child is stunted with a mother who is overweight/obese or when a child is overweight with a mother who is underweight^(14)^. We defined DBM as child stunting and maternal overweight/obesity in the same household, as it is the most prevalent and well-studied measure for assessing household-level DBM in LMIC^(14,22)^. A child was considered stunted if their height-for-age Z-score was below minus two standard deviations (–2 sd) from the 2006 WHO Child Growth Standards median Z-score^(36)^. A mother was considered overweight if her BMI was 25 kg/m^2^ or higher^(37)^. The DBM variable was coded 1 if a child was stunted and the mother was overweight and 0 otherwise.

### Household economic status

Household economic affluence was measured using the DHS wealth index^(33,34)^. The DHS wealth index is a composite measure of a household’s cumulative living standard, constructed using household-level information on ownership of selected assets, such as television and bicycles, materials for housing construction and type of water access and sanitation facilities^(38)^. It is one of the most useful indicators of household financial well-being in LMIC where it is difficult to obtain reliable data on household income from surveys^(33,34)^. This is because a significant portion of the population in LMIC do not receive market-level transactions and engage in significant home production^(21)^. A continuous measure of relative wealth (i.e. wealth index factor score) was assessed for each household using principal component analysis^(33,34)^. Based on the distribution of the wealth index factor score in the whole survey sample of the 2015–2016 Tanzania DHS, all households were categorised into quintiles^(33,34)^.

### Dietary diversity

In the 2015–2016 Tanzania DHS, training of field staff on the nutritional survey was provided by the trainers from the Ifakara Health Institute and Tanzania Food and Nutrition Centre, with support from the Inner City Fund International^(34)^. Mothers were asked if the child was receiving breastmilk and provided a 24-h recall of foods and food groups given to their children^(39)^. Data were collected on the following foods and beverages that the child had consumed the previous day: juice; tinned, powdered or fresh milk; formula milk; fortified baby food (cerelac, etc.); other porridge/gruel; soup/clear broth; other liquids; chicken, duck, or other birds; bread, noodles, other grains; potatoes, cassava, tubers; eggs; meat (beef, pork, lamb, chicken, etc.); pumpkin, carrots, squash; dark green leafy vegetables; mangoes, papayas, other vitamin A fruits; any other fruits; liver, heart, other organ meat; fish or shellfish; beans, peas, lentils, nuts; cheese, yogurt, other milk products; oil, fats, butter, products made of them and other solid/semi-solid food. Eight food groups were defined following the WHO/UNICEF Infant and Young Child Feeding practices guidelines^(39,40)^: (1) breastmilk; (2) grains, roots and tubers; (3) legumes and nuts; (4) dairy products (infant formula, milk, yogurt and cheese); (5) flesh foods (meat, fish, poultry and liver/organ meats); (6) eggs; (7) fruits and vegetables rich in vitamin A and (8) other fruits and vegetables^(41)^.

Dietary diversity is a commonly used indicator of diet quality estimated using the number of different food groups consumed within over a given reference period^(35)^. For each child, a dietary diversity score was computed by counting the number of consumed food groups (ranging from zero to eight). Minimum dietary diversity was defined as having a dietary diversity score > 5, according to the 2021 WHO/UNICEF Infant and Young Child Feeding practices guideline^(40)^ and the DHS statistics guide^(39)^. We used minimum dietary diversity for children as a proxy indicator at the household level since data on the mothers’ diet were not available. Mother–child pairs were categorised into two groups: achieving and not achieving minimum dietary diversity.

### Covariates

We considered the following demographic and socio-economic covariates that may affect both household economic status and the presence of DBM: the mother’s age (in years), education (no completed education, completed primary education or completed secondary education and above), marital status (never married, currently married and formerly married), place of residence (urban or rural), number of children in the household, child’s age (in months) and sex (male or female) and the number of household members.

### Statistical analysis

All statistical analyses were performed using the SAS software (version 9.4; SAS Institute) and R version 4.3.0 (R Foundation for Statistical Computing). All analyses were weighted using sampling weights, which considered the stratified cluster sampling design and non-response rate. The prevalence of DBM by region was illustrated as a choropleth map, and regional differences were tested using χ^2^ tests. We summarised the sample characteristics according to the wealth index quintiles among non-achievers and achievers of minimum dietary diversity. Descriptive statistics were presented as weighted means and SE for continuous variables and weighted frequencies (%) and their SE for categorical variables. We tested the trends in the sample characteristics across quintiles of wealth index using logistic regression model for categorical variables and linear regression model for continuous variables.

We built logistic regression models for stratified cluster sampling to assess the OR and 95 % CI of DBM according to the wealth index levels. Given that there were too few cases of DBM among mother–child pairs who achieved minimum dietary diversity in the poorest group to build logistic regression models, we merged the poorest group with the poorer group. We used both a continuous estimate of the wealth index score and groups of the wealth index as independent variables in separate models. First, we performed unadjusted analyses. We then adjusted the models for all covariates as mentioned above. We tested the trend in the association between the wealth index and DBM by assigning ordinal numbers (0, 1, 2 and 3) to the wealth index categories, treating it as a continuous variable. Based on the hypothesis that the household wealth index might exhibit varying associations with DBM depending on the presence of minimum dietary diversity, we initially tested the heterogeneity in the associations between the two groups of minimum dietary diversity. This was achieved by adding a multiplicative interaction term (minimum dietary diversity × household wealth index). We tested this interaction effect using the likelihood ratio test by comparing the log-likelihood of the model containing the interaction term and that of the model not containing the interaction term. We conducted primary analyses separately for non-achievers and achievers of minimum dietary diversity. We performed a restricted cubic spline analysis without assuming a linear association between the wealth index score and the DBM to visualise the shape of this association. We placed four knots at the 20th (the reference), 40th, 60th and 80th percentiles of the wealth index score. Collinearity between independent variables was checked using the variance inflation factor test.

We performed the following sensitivity analyses: (1) additionally adjusting for region to account for the potential confounding effect in which the association between household wealth and DBM is attributed to regional differences only and (2) removing the variable of place of residence from the covariates to address the potential collinearity between place of residence and the household wealth index (with a variance inflation factor value = 2·3). Statistical tests were two-sided, and a *P* value for the interaction term < 0·05 was considered statistically significant.

## Results

**Fig. 1:**
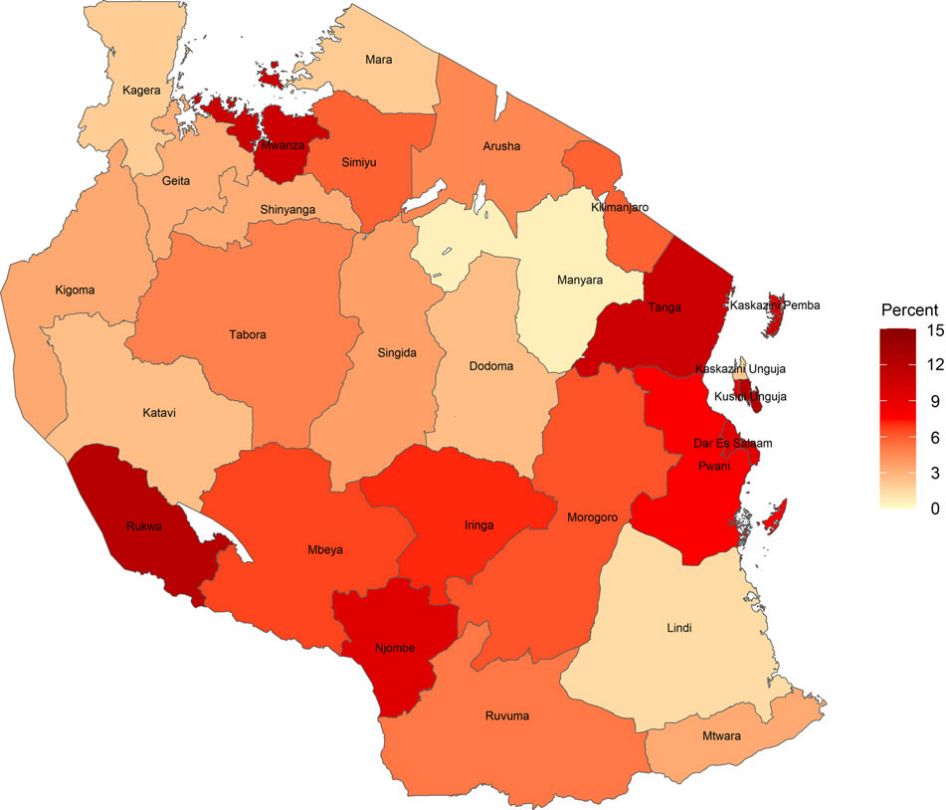
The estimated prevalence of double burden of malnutrition in Tanzania by region

The estimated prevalence (s e) of DBM was 5·6 % (0·6). The prevalence (se) of child stunting was 31·1 % (1·2), and maternal overweight was 21·4 % (1·0). Figure 1 shows the regional distribution of the DBM prevalence in Tanzania, which ranged from the lowest rate of 0·6 % in Manyara to the highest rate of 12·2 % in Kusini Unguja, with significant regional differences (*P* = 0·03). In total, 21·8 % (1·0) of mother–child pairs achieved minimum dietary diversity. The estimated prevalence (se) of DBM was 5·4 % (0·6) among non-achievers of minimum dietary diversity and 6·7 % (1·2) among achievers of minimum dietary diversity.

Table 1 shows the characteristics of mother–child pairs according to the quintiles of the household wealth index among non-achievers and achievers of minimum dietary diversity. In both groups, households with a higher wealth index were more likely to have mothers with higher education, had few living children and household members and lived in urban areas. They were also more likely to have children with lower height-for-age and mothers with higher BMI. In non-achievers, households with a higher wealth index were more likely to have mothers who were younger and never or formerly married.

**Table 1:** Characteristics of mother–child pairs according to the household wealth index among non-achievers and achievers of minimum dietary diversity

	Non-achievers of minimum dietary diversity						Achievers of minimum dietary diversity					
	Poorest	Poorer	Middle	Richer	Richest	*P* trend	Poorest	Poorer	Middle	Richer	Richest	*P* trend
Number of mother–child pairs	544	498	441	352	320		89	79	97	171	176	
Age of mother (se), years												
Mean	28·3	28·1	27·9	27·0	27·4	0·03	27·8	27·9	28·5	27·2	28·9	0·35
se	0·3	0·4	0·4	0·4	0·5		0·9	0·8	0·8	0·6	0·5	
Age of child (se), months												
Mean	14·0	13·9	14·0	14·1	14·4	0·32	14·1	13·7	15·3	14·7	14·4	0·54
se	0·3	0·3	0·3	0·3	0·4		0·5	0·6	0·4	0·4	0·4	

	%	se	%	se	%	se	%	se	%	se		%	se	%	se	%	se	%	se	%	se	
Sex of child (female), % (se)	49·9	2·5	49·3	2·7	48·3	3·0	49·3	2·9	46·0	3·5	0·91	42·6	6·9	57·9	7·0	47·1	6·0	55·3	4·8	46·7	4·4	0·30
Mother’s education, % (se)											<0·001											<0·001
No completed education	36·3	3·0	28·3	2·7	18·9	2·1	11·8	2·0	1·3	0·5		26·0	5·4	35·4	6·2	5·1	2·4	6·7	2·3	0·8	0·5	
Primary education	59·2	3·1	65·9	2·6	70·9	2·5	68·8	2·5	51·8	3·7		70·0	5·6	60·3	6·3	84·8	3·8	61·6	4·4	38·0	4·6	
Secondary education or above	4·5	1·1	5·8	1·3	10·2	1·8	19·4	2·1	46·9	3·7		4·0	1·9	4·3	2·3	10·1	3·1	31·7	4·2	61·2	4·6	
Current marital status, % (se)											<0·001											0·50
Never married	3·1	0·8	4·6	1·1	8·3	1·7	17·8	2·4	14·5	2·6		2·3	1·8	9·7	5·1	7·9	2·7	9·8	2·8	5·2	1·6	
Currently married	62·6	2·9	61·9	3·1	51·2	3·1	49·2	3·1	62·3	4·0		70·1	5·8	54·1	6·0	63·4	5·5	62·8	4·7	66·3	4·3	
Formerly married	34·3	2·8	33·5	3·0	40·5	2·8	33·0	3·2	23·2	3·3		27·6	5·6	36·2	5·6	28·7	5·0	27·4	4·1	28·4	4·2	
Number of living children in the household (se)																						
Mean	3·9		3·6		3·5		2·7		2·2		<0·001	3·5		3·7		3·4		2·8		2·2		<0·001
se	0·1		0·1		0·1		0·1		0·1			0·2		0·3		0·2		0·1		0·1		
Number of household members (se)																						
Mean	8·5		7·3		7·4		6·7		6·7		<0·001	8·8		8·0		6·6		6·1		5·8		<0·001
se	0·4		0·3		0·3		0·3		0·2			0·8		0·6		0·3		0·3		0·2		
Place of residence (urban), % (se)	5·2	1·9	1·9	0·7	7·8	1·5	50·9	3·7	85·1	3·0	<0·001	6·8	2·6	2·7	2·1	10·1	3·4	40·5	4·6	88·3	2·6	<0·001
Food groups being fed (yes), % (se)																						
Breastmilk	85·5	1·9	81·7	1·9	81·5	2·0	76·8	2·6	70·5	3·1	<0·001	98·1	1·6	96·9	2·0	94·8	2·3	86·7	3·0	87·8	3·2	0·01
Grains, roots and tubers	81·3	2·0	85·0	1·9	88·9	1·9	91·0	1·8	87·7	2·4	0·01	98·9	1·1	96·2	2·3	96·6	2·0	99·5	0·5	97·8	1·6	0·43
Legumes and nuts	21·6	2·3	26·9	2·6	31·4	2·7	33·5	3·1	23·8	3·1	0·01	72·5	5·7	79·6	4·7	71·7	5·7	72·2	4·1	67·3	3·7	0·51
Dairy products	22·1	2·0	15·1	2·1	8·8	1·7	9·4	1·6	24·7	3·2	<0·001	50·7	6·4	37·2	6·0	39·0	6·0	43·3	4·9	48·6	4·5	0·45
Flesh foods	16·6	2·1	15·1	1·9	20·1	2·4	28·1	2·6	38·7	3·4	<0·001	45·5	6·3	63·0	6·7	62·6	5·5	72·7	4·0	76·0	3·9	<0·001
Eggs	1·9	0·6	1·8	0·7	2·1	0·7	2·9	1·1	7·4	2·1	<0·001	10·0	3·3	14·5	4·2	20·9	4·8	24·5	4·3	34·7	5·3	<0·001
Vitamin A rich fruits and vegetables	53·0	3·0	57·3	2·9	58·7	3·0	54·4	3·5	57·3	3·3	0·58	96·1	2·7	91·1	3·7	92·4	2·8	91·7	2·3	95·3	1·7	0·58
Other fruits and vegetables	11·2	1·6	10·2	1·5	11·1	2·1	10·6	1·9	15·2	2·5	0·48	38·2	6·3	43·7	6·7	49·7	5·7	54·0	5·1	59·3	4·3	0·05
BMI of mother (se), kg/m^2^																						
Mean	21·4		21·7		22·2		22·9		25·2		<0·001	21·1		22·0		22·5		24·5		26·8		<0·001
se	0·1		0·1		0·2		0·2		0·4			0·3		0·4		0·4		0·6		0·6		
Height-for-age Z-score of child (se)																						
Mean	−1·4		−1·4		−1·5		−1·3		−1·0		0·02	−1·5		−1·4		−1·6		−1·3		−0·7		<0·001
se	0·1		0·1		0·1		0·1		0·1			0·2		0·2		0·2		0·1		0·1		

Values are frequency (%) or mean. Frequencies, means and SEs are weighted using the sampling weights.

**Fig. 2:**
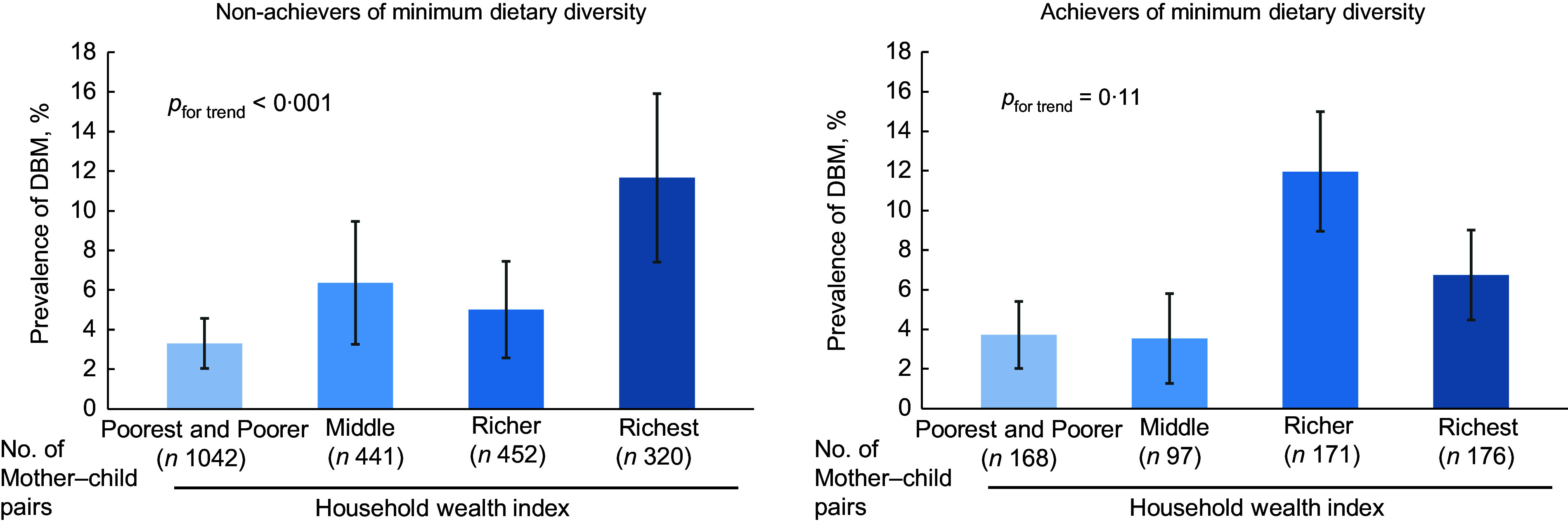
The estimated prevalence and 95 % CI of DBM according to the household wealth index levels among non-achievers and achievers of minimum dietary diversity. The error bar denotes 95 % CI of the prevalence. The poorest group was merged with the poorer group as there was only one case of DBM in the poorest group among those who achieved minimum dietary diversity. *The trend of the association was assessed by assigning ordinal numbers to each group of the household wealth index and modelling this variable as a continuous variable. DBM, double burden of malnutrition

Figure 2 shows the prevalence of DBM according to the household wealth level among non-achievers and achievers of minimum dietary diversity. The prevalence of DBM showed a statistically significant increase with increasing household wealth index among non-achievers of minimum dietary diversity. However, this was not observed among achievers. The DBM prevalence reached a plateau in the richer group and then decreased in the richest group.

Table 2 shows the associations of household wealth index with DBM significantly differed by minimum dietary diversity, with *P*_for interaction_ = 0·006. The multivariable-adjusted odds of DBM in non-achievers of minimum dietary diversity were approximately two times higher for both middle and richer groups and more than five times higher in the richest group, as compared with the poorest/poorer groups (*P*_for trend_ < 0·001). However, the multivariable-adjusted odds of DBM among achievers were not statistically different in the middle and the richest groups, but they were approximately five times higher in the richer group. Similar results were observed when modelling the continuous variable of the wealth index score (mean (sd): 0·16 (0·94)), with an OR (95 % CI) per unit increase in the wealth index score of 2·10 (1·36, 3·25) among non-achievers of minimum dietary diversity and an OR (95 % CI) of 1·38 (0·76, 2·54) among achievers. Restricted cubic spline analyses showed a similar shape association between the wealth index score and DBM among non-achievers and achievers of minimum dietary diversity (see online supplementary material, Supplemental Fig. S2). The prevalence of child stunting decreased as household wealth increased, especially among achievers of minimum dietary diversity. However, the prevalence of maternal overweight increased with the household wealth levels in both non-achievers and achievers of minimum dietary diversity (see online supplementary material, Supplemental Fig. S3).

**Table 2:** Associations between household wealth index and the double burden of malnutrition among non-achievers and achievers of minimum dietary diversity (MDD)

	Unadjusted analyses							Adjusted analyses						
	Non-achievers of MDD (*n* = 2255)		*P* value	Achievers of MDD (*n*= 612)		*P* value	*P*_for interaction_ _between wealth index and MDD_	Non-achievers of MDD (*n* = 2255)		*P* value	Achievers of MDD (*n* = 612)		*P* value	*P*_for interaction_ _between wealth index and MDD_
	OR	95 % CI		OR	95 % CI			OR	95 % CI		OR	95 % CI		
Quintiles of wealth index														
Poorest and poorer*	1·00 (ref)			1·00 (ref)			0·004	1·00 (ref)			1·00 (ref)			0·006
Middle	1·98	1·08, 3·65	0·03	0·95	0·19, 4·66	0·95		2·0	1·09, 3·67	0·02	0·84	0·20, 3·59	0·81	
Richer	1·54	0·81, 2·93	0·19	3·52	1·18, 10·50	0·02		1·9	0·90, 4·18	0·09	4·74	1·23, 18·30	0·03	
Richest	3·86	2·18, 6·82	<0·001	1·87	0·57, 6·11	0·30		5·7	2·17, 15·36	<0·001	2·03	0·33, 12·46	0·44	
*P*_for trend_ †	<0·001			0·11				<0·001			0·16			
Wealth index score (continuous, per one unit increment)														
	1·62	1·31, 2·01	<0·001	1·25	0·90, 1·73	0·19		2·10	1·36, 3·25	<0·001	1·38	0·76, 2·54	0·29	

DBM, double burden of malnutrition. Adjusted models were adjusted for mother’s age (in years), education (no completed education, completed primary education or completed secondary education and above), marital status (never married, currently married and formerly married), place of residence (urban or rural), number of children in the household, child’s age (in months) and sex (male or female) and number of household members.

* The poorest group was merged with the poorer group as there was only 1 case of DBM in the poorest group among those who achieved minimum dietary diversity to build the logistic regression model.

† Trend association was assessed by assigning ordinal numbers to each group of household wealth index and modelling this variable as a continuous variable.

In the sensitivity analyses, the results minimally changed after further adjustment for regions (see online supplementary material, Supplemental Table S1), or without adjustment for the place of residence (see online supplementary material, Supplemental Table S2).

## Discussion

This analysis demonstrated that the prevalence of household-level DBM varied regionally and was unequally distributed across levels of household wealth. Inequalities in DBM across household wealth levels were moderated by minimum dietary diversity. Richer households had higher odds of DBM, but this association was less pronounced in mother–child pairs achieving minimum dietary diversity. Our findings suggest that household wealth increased DBM in Tanzania; however, dietary diversity could potentially mitigate this negative impact. This study is one of the few attempts to examine the economic inequalities in DBM at the household level in Tanzania by considering the moderating role of dietary diversity in these inequalities.

Our observation of the prevalence of DBM at the household level in Tanzania is comparable with the results from analyses of LMIC (5·6 % *v*. 6·0 %)^(22)^. However, our observations indicate relatively higher rates of DBM compared with LMIC in Asia, where the prevalence was mostly < 1 %^(21)^. This disparity may be primarily driven by the high prevalence of maternal overweight or obesity in Tanzania (31·7 % in 2018)^(12)^. We also observed regional differences in the prevalence of DBM. DBM tended to be disproportionately concentrated in regions with relatively higher economic development levels, such as Kusini Unguja, Mwanza, Tanga and Dar es Salaam. This observation could be partly explained by the fact that economic growth of an entire area might exacerbate the DBM prevalence^(21,22)^. Taken together, our findings suggest the importance of accounting for regional differences including varying economic development levels, when addressing DBM in Tanzania.

Our findings on the negative impact of household economic affluence on household-level DBM in Tanzania agree with findings from previous limited analysis in fifty-five LMIC^(22)^ and eleven LMIC in Asia^(21)^, as well as other analyses using nationally representative data^(19,20)^. In contrast, some analyses showed no or opposite direction of the association^(17,18)^. Of note, no previous analysis has examined the interaction between household economic affluence and dietary diversity on DBM. This study expands on existing evidence regarding the adverse impact of household wealth on DBM and demonstrated that dietary diversity could potentially alleviate these negative impacts. This moderating effect of dietary diversity could be driven by the observed dramatic decrease in the child stunting rate among the richest households that embraced a minimum level of dietary diversity. A more diverse diet is highly correlated with higher micronutrient intake among children, thus helping prevent child stunting^(28)^. The dramatic decrease in child stunting in the richest households could be attributed to the fact that their mothers were more likely to have a higher level of nutritional literacy, in addition to more food expenditure to sustain a high overall diet quality for their children^(42)^. We also observed a persistent increase in maternal overweight as household wealth increased, even among the group that achieved minimum dietary diversity. This result indicates that affluent Tanzanian women have a high level of total energy intake, regardless of dietary diversity. This finding could be partly explained by the cultural beliefs held by Tanzanian women that associate overweight/obesity with beauty and consider it a symbol of success in life^(43)^. Our findings support that dietary diversity might be an underrated action target for addressing DBM^(23,24)^.

A recent Lancet Commission advocated double-duty actions to simultaneously address different forms of malnutrition, aligning with the United Nations’ SDG and global nutrition targets^(23,24)^. Our findings indicate that double-duty actions promoting dietary diversity for children while simultaneously reducing total energy intake among mothers could be an effective strategy to address DBM in Tanzania. Additionally, the design of such double-duty actions should consider the uneven impact of economic affluence, as well as cultural and regional differences.

This study used a large nationally representative sample and employed robust methodological approaches, including interactions between household wealth and dietary diversity and restricted cubic splines to avoid assuming linear associations. Our results remained robust under different sensitivity analyses. However, this study has several limitations. The cross-sectional nature precludes causal inferences. We did not include children aged 2 years and older because the DHS employed the WHO-designed indicator of minimum dietary diversity specifically for children 6–23 months^(35)^. Future studies should validate our findings among children aged 24–59 months and their mothers in LMIC^(44)^. This study was also limited by the lack of data on mother’s diet. In Tanzania, there is a food culture in which women and children eat from the same pot^(27)^, indicating that what mothers eat is strongly related to what their children eat^(45)^. Nevertheless, we could not rule out the possibility of misclassification of mother–child pairs’ minimum dietary diversity, which may have led to an underestimation of the moderating effect of dietary diversity on DBM. There is a chance that the statistical power could be inadequate for the analysis among achievers of minimum dietary diversity. However, we observed significantly higher odds of DBM among non-achievers compared with their counterparts. Thus, this is unlikely to alter our conclusion. Although we used the most prevalent measure of DBM, other forms of DBM exist and may exhibit different associations with household wealth. The wealth index is a country-specific and relative measure of household wealth affluence. We urge caution when generalising our findings to other countries.

In conclusion, the prevalence of household-level DBM was unequally distributed across regions of Tanzania and increased with higher household wealth. However, the association between household wealth and DBM was mitigated by dietary diversity levels. Our findings highlight the importance of increasing dietary diversity to address the negative impact of household wealth on DBM in Tanzania.

## Supporting information

Chen et al. supplementary material Chen et al. supplementary material

## Supplementary material

For supplementary material accompanying this paper visit https://doi.org/10.1017/S136898002400106X
