# Supplementary material for: Dietary diversity moderates household economic inequalities in the double burden of malnutrition in Tanzania
Source: Public Health Nutr. 2024 May 16;27(1):e141. doi: 10.1017/S136898002400106X (PMC11374552; doi:10.1017/S136898002400106X)
Supplement: Chen et al. supplementary material [file S136898002400106Xsup001.pdf]

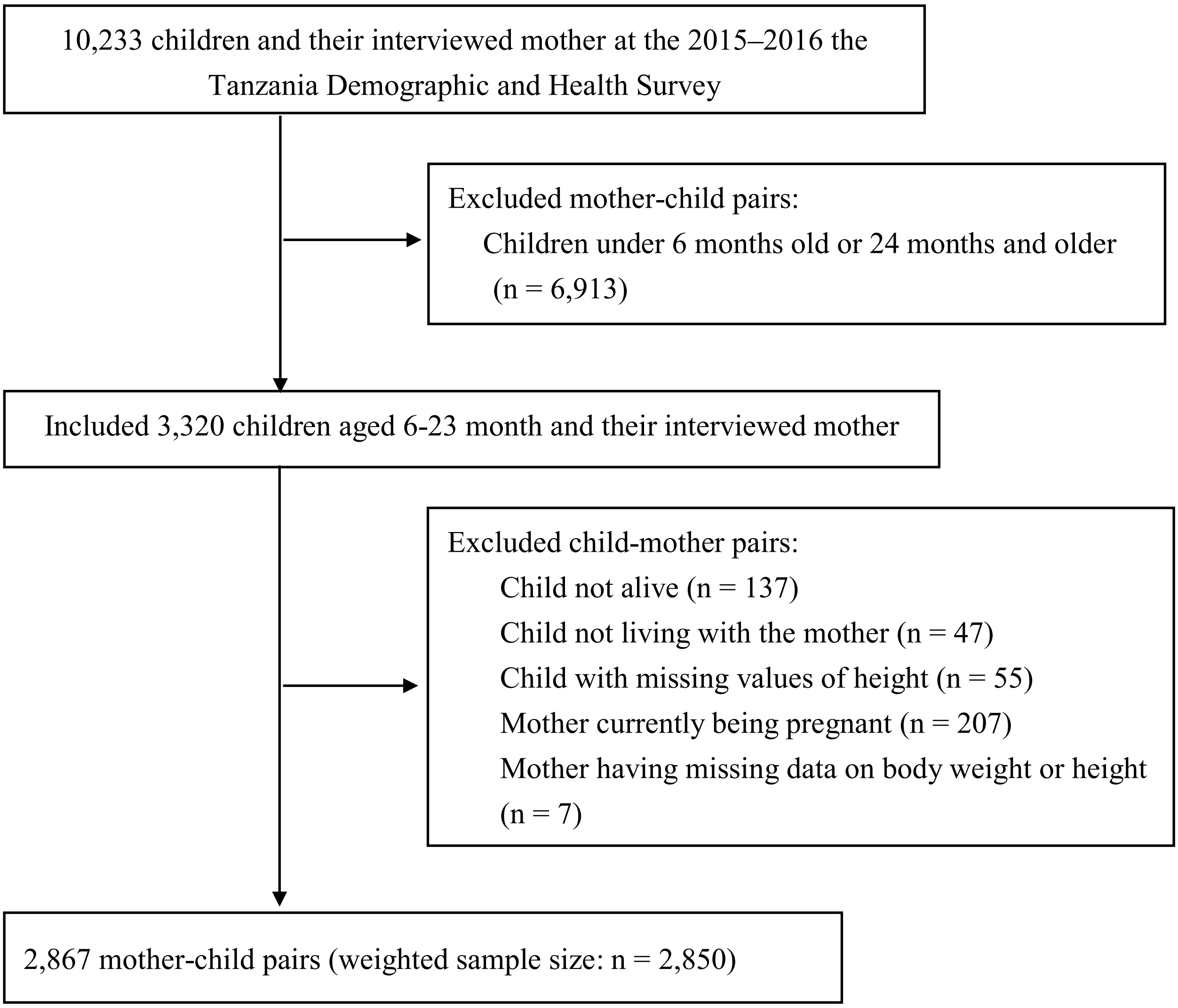

Supplemental Figure S1 Flowchart of the study sample

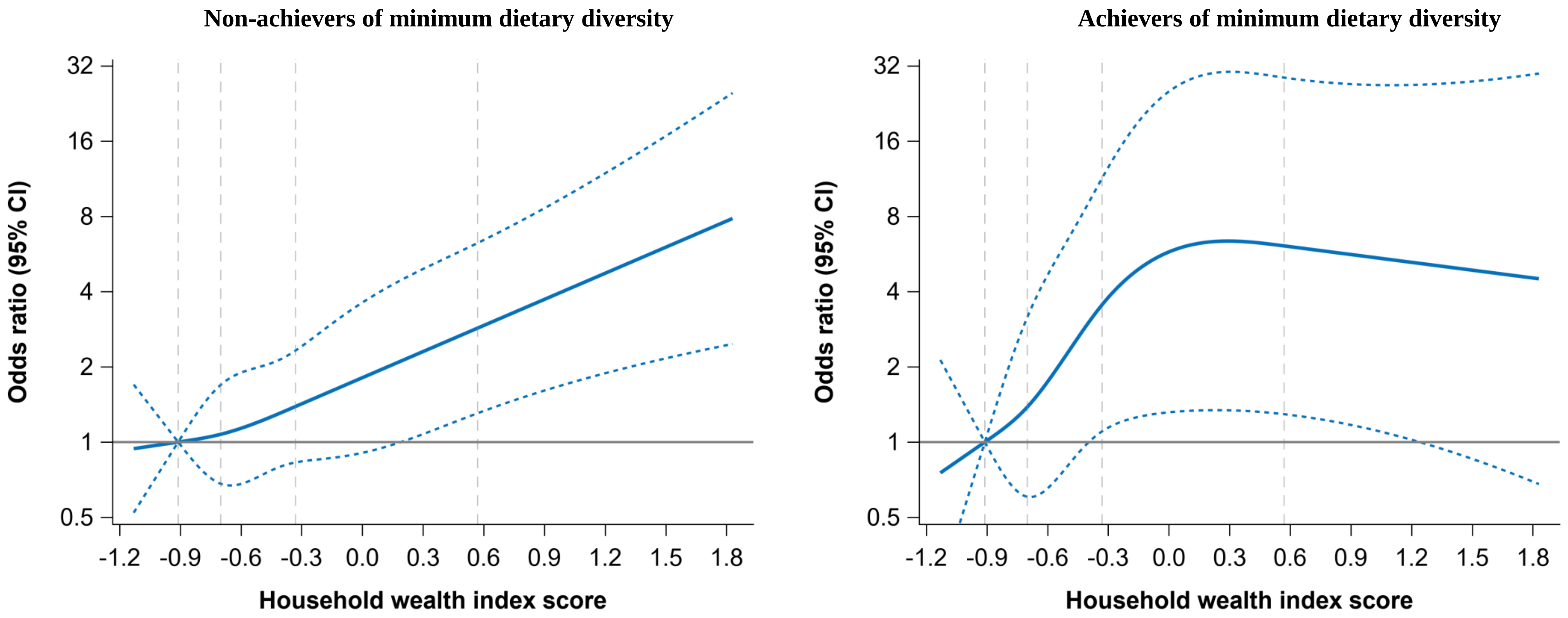

Supplemental Figure S2. Restricted cubic splines for the association between household wealth index score and DMB among non-achievers and achievers of minimum dietary diversity

Solid lines represent the ORs; dashed lines represent the 95% CI. Knots were placed at the 20th, 40th, 60th, and 80th percentiles of the household wealth index score. A reference point was set at 20th percentile. Vertical dashed lines indicate the 20th, 40th, 60th, and 80th percentiles of the household wealth index score. The model was adjusted for mother's age (in years) and education (no completed education, completed primary education, or completed secondary education and above), marital status (never married, currently married, formerly married), place of residence (urban or rural), number of children in the household, child's age (in months) and sex (male or female), and number of household members.

CI=confidence interval; DBM=double burden of malnutrition; OR=Odds ratio.

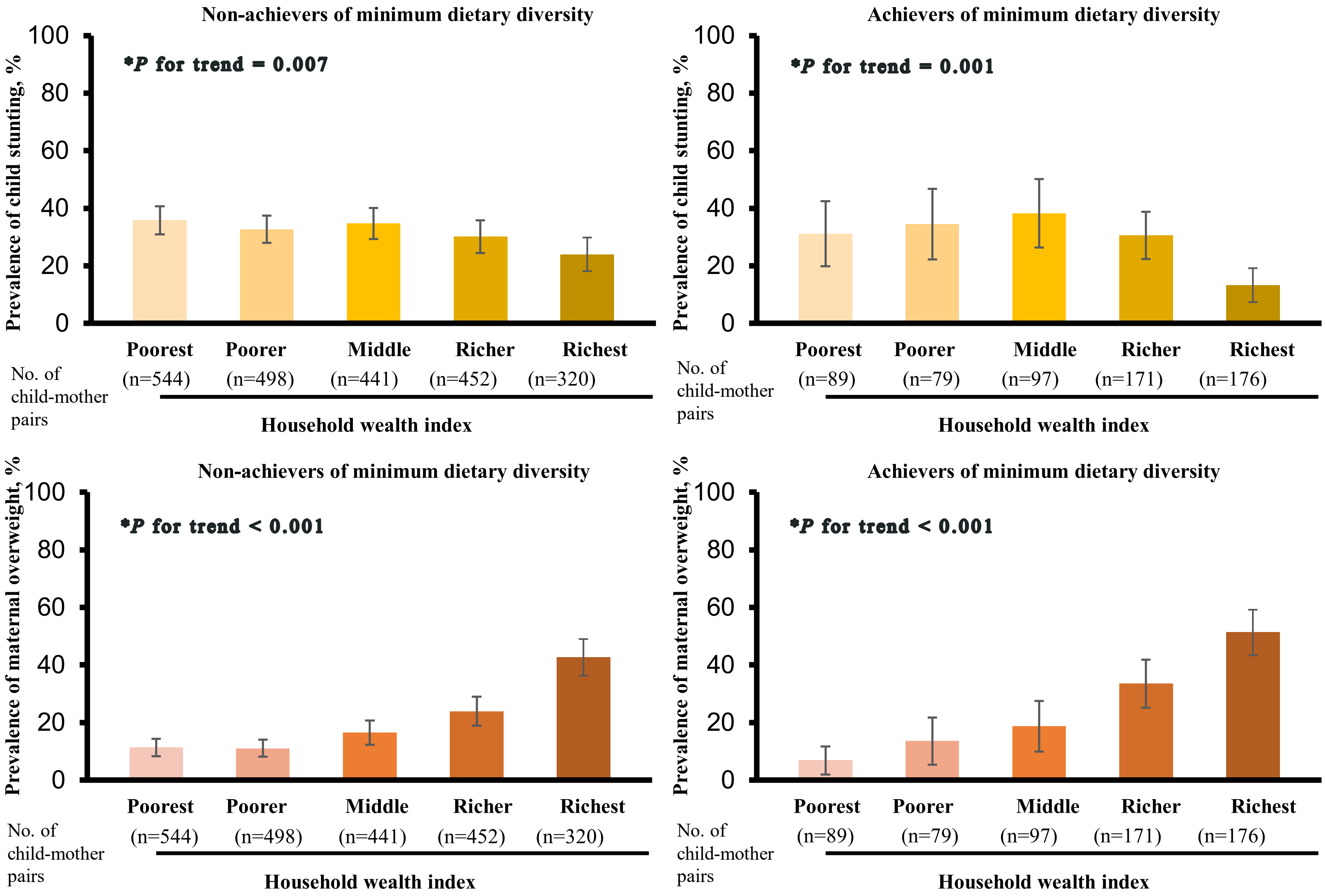

Supplemental Figure S3. The estimated prevalence and 95% confidence interval of DBM among non-achievers and achievers of minimum dietary diversity

*Trend association was assessed by assigning ordinal numbers to each group of the household wealth index and modelling this variable as a continuous variable. DBM=double burden of malnutrition.

Supplemental Table S1 Associations between the household wealth index and the double burden of malnutrition among non-achievers and achievers of minimum dietary diversity (MDD) with additional adjustment for region.

	Without MDD (n=2,278)	*p* value	With MDD (n=612)	*p* value	*p* for interaction between wealth index and MDD
	OR (95% CI)		OR (95% CI)		
Quintiles of wealth index					
Poorest & poorer*	1.00 (ref)		1.00 (ref)		
Middle	2.04 (1.09-3.82)	0.03	0.53 (0.10-2.72)	0.45	0.005
Richer	1.65 (0.71-3.79)	0.24	4.19 (1.04-16.86)	0.04	
Richest	4.46 (1.67-11.89)	0.003	1.31 (0.15-11.51)	0.81	
p for trend†	0.005		0.19		
Wealth index score (continuous, per one unit increment)					
	1.90 (1.23-2.94)	0.004	1.50 (0.59-3.82)	0.39	

CI=confidence interval; DBM=double burden of malnutrition; OR=odds ratio. Models were adjusted for mother's age (in years), education (no completed education, completed primary education, or completed secondary education and above), marital status (never married, currently married, formerly married), place of residence (urban or rural), number of children in the household, child's age (in months) and sex (male or female), number of household members, and region.

*The poorest group was merged with the poorer group as there was only 1 case of DMB in the poorest group among achievers of minimum dietary diversity to build the logistic regression model.

†Trend association was assessed by assigning ordinal numbers to each group of the household wealth index and modeling this variable as a continuous variable.

Supplemental Table S2 Associations between the household wealth index and the double burden of malnutrition among non-achievers and achievers of minimum dietary diversity (MDD) without adjustment for place of residence.

	Without MDD (n=2,278)	*p* value	With MDD (n=612)	*p* value	*p* for interaction between wealth index and MDD
	OR (95% CI)		OR (95% CI)		
Quintiles of wealth index					
Poorest & poorer*	1.00 (ref)		1.00 (ref)		
Middle	1.98 (1.09-3.63)	0.03	0.84 (0.19-3.62)	0.81	0.005
Richer	1.60 (0.82-3.28)	0.16	4.12 (1.09-15.66)	0.04	
Richest	4.23 (1.93-9.25)	<0.001	1.36 (0.30-6.25)	0.69	
p for trend†	0.001		0.41		
Wealth index score (continuous, per one unit increment)					
	1.71 (1.28-2.29)	<0.001	1.08 (0.70-1.68)	0.72	

CI=confidence interval; DBM=double burden of malnutrition; OR=odds ratio. Models were adjusted for mother's age (in years), education (no completed education, completed primary education, or completed secondary education and above), marital status (never married, currently married, formerly married), number of children in the household, child's age (in months) and sex (male or female), and number of household members.

*The poorest group was merged with the poorer group as there was only 1 case of DMB in the poorest group among achievers of minimum dietary diversity to build the logistic regression model.

†Trend association was assessed by assigning ordinal numbers to each group of the household wealth index and modeling this variable as a continuous variable.
